# Attenuation of **β**-Amyloid-Induced Oxidative Cell Death by Sulforaphane via Activation of NF-E2-Related Factor 2

**DOI:** 10.1155/2013/313510

**Published:** 2013-06-20

**Authors:** Chan Lee, Gyu Hwan Park, Seong-Ryong Lee, Jung-Hee Jang

**Affiliations:** ^1^Department of Pharmacology, School of Medicine, Keimyung University, Daegu 704-701, Republic of Korea; ^2^Research Institute of Pharmaceutical Sciences, College of Pharmacy, Kyungpook National University, Daegu 702-701, Republic of Korea

## Abstract

*β*-amyloid peptide (A*β*), a major component of senile plaques, plays important roles in neuropathology of Alzheimer's disease (AD). An array of *in vitro* and *in vivo* data indicates that A*β*-induced neuronal death is mediated by oxidative stress. In this study, we aimed to investigate effects of sulforaphane (SUL), an isothiocyanate in cruciferous vegetables, on A*β*-induced oxidative cell death in SH-SY5Y cells. Cells treated with A*β*_25–35_ exhibited decreased cell viability and underwent apoptosis as determined by MTT assay and TUNEL, respectively. A*β*_25–35_-induced cytotoxicity and apoptotic characteristics such as activation of c-JNK, dissipation of mitochondrial membrane potential, altered expression of Bcl-2 family proteins, and DNA fragmentation were effectively attenuated by SUL pretreatment. The antiapoptotic activity of SUL seemed to be mediated by inhibition of intracellular accumulation of reactive oxygen species and oxidative damages. SUL exerted antioxidant potential by upregulating expression of antioxidant enzymes including **γ**-glutamylcysteine ligase, NAD(P)H:quinone oxidoreductase-1, and heme oxygenase-1 via activation of NF-E2-related factor 2(Nrf2). The protective effect of SUL against A*β*_25–35_-induced apoptotic cell death was abolished by siRNA of Nrf2. Taken together, the results suggest that pharmacologic activation of Nrf2 signaling pathway by SUL might be a practical prevention and/or protective treatment for the management of AD.

## 1. Introduction

Alzheimer's disease (AD) is one of the most common forms of senile dementia, characterized by progressive loss of memory and decline of cognitive functions due to neuronal death in the brain. There are two classical pathological hallmarks of AD [1]. One is extraneuronal accumulation of amyloid plaques composed of *β*-amyloid peptide (A*β*), which is produced by proteolytic cleavage from amyloid precursor protein (APP) with sequential actions of *β*- and *γ*-secretases. The other is intraneuronal deposits of neurofibrillatory tangles (NFT) consisting of hyperphosphorylated tau protein generated by actions of upstream kinases such as glycogen synthase kinase-3*β* (GSK-3*β*) and cyclin-dependent kinase 5 (CDK5). Therefore, treatments for AD have been developed based on these two molecular approaches [2]. A*β*-based therapies include utilizing *β*-  or *γ*-secretases inhibitors, A*β* aggregation blockers, and A*β* catabolism inducers. Tau-based therapies make an advantage of upstream kinase inhibitors, microtubule stabilizers, and tau catabolism inducers.

However, pathogenesis of AD appears to be multifactorial events, whereby genetic as well as environmental factors, oxidative stress, depletion of endogenous antioxidants, altered ion levels, inflammation, disruption in neurotransmission, synaptic dysfunction, and neuronal cell death operate sequentially or in parallel [3]. Among them we have focused on A*β*-induced oxidative damages and neuronal cell death as one of the major causes of AD pathology. Oxidative stress has been proposed to be an important factor in the development and progression of AD and contributes to A*β* aggregation and NTF formation as well [4]. Reactive oxygen species (ROS) can be derived from diverse cellular sources, among which are enzymatic reactions, mitochondrial deterioration, and imbalance in redox transition metal ions. The excessive production and accumulation of ROS by A*β* can cause functional and structural changes in critical macromolecules leading to lipid peroxidation, protein oxidation, and DNA cleavage and altered signal transduction [5]. The levels of molecular markers for lipid peroxidation (HNE, isoprostanes, etc.) and oxidation of proteins (carbonyls) and DNAs (8-OHdG) are reported to be elevated in the brains or cerebrospinal fluid of patients with AD [6].

Given the involvement of A*β*-induced oxidative stress in the etiology and pathology of AD, one of the promising approaches to preventive interventions for AD includes antioxidant therapy by inhibiting the detrimental effects of excess ROS through induction of endogenous antioxidant enzymes. Particularly, many studies highlighted natural phytochemicals derived from medicinal herbs and foods as potential candidates which can protect neurons against various toxic compounds and exert beneficial effects on neuronal cells [7, 8]. Sulforaphane (4-methylsulfinylbutyl isothiocyanate, SUL) is a naturally occurring isothiocyanate present in cruciferous vegetables, such as broccoli, cabbage, and cauliflower and has been shown to exhibit anticarcinogenic, anti-inflammatory, antioxidant, chemopreventive, and cytoprotective properties [9, 10]. Recently, it has been reported that SUL can penetrate blood brain barrier and exert neuroprotective effects in diverse *in vitro* cell culture and *in vivo* animal models of neurological disorders [11, 12]. SUL has been reported to attenuate microglia-induced inflammation in hippocampus of LPS-treated mice and BV-2 microglia cells [13]. In addition, SUL protected against oxidative stress induced by hypoxia-ischemic injury [14], oxygen and glucose deprivation [15], 6-hydroxydopamine (6-OHDA) [16], superoxide [17], hydrogen peroxide (H_2_O_2_), and glutamate [18]. However, there has been no direct evidence demonstrating that the protective effect of SUL against A*β*-induced oxidative damage and cell death as yet.

Therefore, in this study we examined whether SUL can suppress A*β*_25–35_-induced oxidative damage and cell death in human neuroblastoma SH-SY5Y cells via augmentation of antioxidant defense capacity by activation of NF-E2-related factor 2 (Nrf2) and the subsequent expression of antioxidant and phase II detoxification enzymes which play key roles in inhibiting ROS production and oxidative damages.

## 2. Materials and Methods

### 2.1. Chemicals and Reagents

A*β*_25–35_  and SUL were purchased from American Peptide (Sunnyvale, CA, USA) and LKT Laboratories, Inc. (St. Paul, MN, USA), respectively. Dulbecco's modified Eagle's medium (DMEM), fetal bovine serum (FBS), and penicillin-streptomycin antibiotic were supplied from Gibco BRL (Grand Island, NY, USA). Tetramethylrhodamine ethyl ester (TMRE) and 2′7′-dichlorofluorescein diacetate (DCF-DA) dyes were the products of Invitrogen Co. (Carlsbad, CA, USA). Anti-phospho-JNK (p-JNK), anti-JNK, anti-Bcl-2, anti-Bax, anti-*γ*-glutamylcysteine ligase (GCL), anti-NAD(P)H:quinone oxidoreductase-1 (NQO-1), and anti-Nrf2 antibodies were obtained from Santa Cruz Biotechnology, Inc (Santa Cruz, CA, USA). Anti-heme oxygenase-1 (HO-1) antibody was provided by Stressgen (Ann Arbor, MI, USA). Anti-4-hydroxynonenal (4-HNE) and anti-phospho-Nrf2 antibodies were supplied from Abcam (Cambridge, MA, USA) and Epitomics, Inc. (Burlingame, CA, USA), respectively. MTT [3-(4,5-dimethylthiazol-2-yl)-2,5 diphenyltetrazolium bromide], anti-actin antibody, and other chemical reagents were purchased from Sigma-Aldrich (St. Louis, MO, USA).

### 2.2. SH-SY5Y Cell Culture

SH-SY5Y cells were maintained in DMEM media containing 10% FBS, penicillin (10000 U), and streptomycin (100 *μ*g/mL) in a 5% CO_2_ incubator at 37°C under a humidified atmosphere. The media were changed every other day. Cells were seeded at an appropriate density according to the each experimental scale.

### 2.3. Cell Viability Assay (MTT Dye Reduction Assay)

Cytotoxicity was determined by the conventional MTT dye reduction assay. Cells were seeded in 48-well plate at a density of 5 × 10^4^ cells/well and incubated with A*β*_25–35_ (15 *μ*M) for 24 h with or without 30 min pretreatment of SUL (1, 2, and 5 *μ*M) or *N*-acetylcysteine (NAC, 0.5 and 1 mM). After treatment, MTT solution (5 mg/mL) was added and further incubated for 2 h at 37°C. The formazan crystals formed in viable cells were extracted with 200 *μ*L of dimethylsulfoxide (DMSO) and the absorbance was measured in a microplate reader at 570 nm (Emax, Molecular Device, CA, USA). Relative cytotoxicity was calculated as percentage of viable cells with respect to the optical density (OD) value of the living cells in the control as 100%.

### 2.4. Measurement of DNA Fragmentation (TUNEL)

For detection of DNA fragmentation, terminal deoxynucleotidyl transferase-mediated dUTP nick end labeling (TUNEL) (Roche diagnostics GmbH, Mannheim, Germany) was performed in SH-SY5Y cells (8 × 10^4^ cells/500 *μ*L in 4-well chamber slide) exposed to 15 *μ*M A*β*_25–35_ for 24 h in the presence or absence of SUL or NAC pretreatment. The slide was rinsed with phosphate-buffered saline (PBS) three times and fixed in 10% neutral buffered formalin solution for 30 min at room temperature (RT). After incubation with 0.3% H_2_O_2_  in methanol for 30 min at RT to inactivate endogenous peroxidase, the slide was further reacted with a permeabilizing solution (0.1% sodium citrate and 0.1% Triton X-100) for 2 min at 4°C. The cells were treated with TUNEL reaction mixture for 1 h at 37°C and then labeled with antidigoxigenin peroxidase for additional 30 min at 37°C. After rinsing with PBS three times, color development was performed with 3,3′-diaminobenzidine (Vector Laboratories, CA, USA). The stained images were examined under a light microscope (Leica Microsystems, Wetzlar, Germany).

### 2.5. Western Bolt Analysis

Cells extracts were prepared by washing cells with PBS and centrifugation at 7,000 g for 5 min. The collected cells were lysed with RIPA buffer (Pierce Biotechnology, Inc., Rockford, IL, USA) and protease inhibitor cocktail tablet (Roche Diagnostics) on ice for 30 min. Protein concentration was quantified by BCA Protein Assay (Pierce Biotechnology). Protein samples were boiled in SDS sample buffer and separated on SDS-PAGE using 10%–12% acrylamide gels. Subsequently, protein samples were transferred onto polyvinylidene fluoride (PVDF, Roche Diagnostics) membranes by transblot electrophoretic transfer for 3 h at a constant current of 300 mA. The nonspecific binding of antibodies was blocked using 5% (w/v) nonfat milk in PBS containing 0.1% Tween-20 (PBST) for 1 h at RT. After blocking, the membranes were probed with the primary antibodies overnight at 4°C. The membranes were washed in PBST three times for 10 min each. The corresponding secondary antibodies were diluted in PBST and reacted with the membranes for 1 h at RT. Finally, immunoreactive bands were visualized by chemiluminescence method (Pierce Biotechnology). The images and relative density of immunoreactive bands were analyzed by using ImageQuant LAS 4000 Multi-Gauge software (Fujifilm, Tokyo, Japan).

### 2.6. Measurement of Mitochondrial Membrane Potential (MMP)

For detection of mitochondrial transmembrane potential in SH-SY5Y cells, TMRE probe was utilized. The cells were seeded at a density of 8 × 10^4^ cells/500 *μ*L in 4-well chamber slide and treated with 15 *μ*M A*β*_25–35_  for 24 h in the absence or presence of SUL or NAC. After treatment, cells were washed with PBS and further incubated with TMRE solution (50 *μ*M in PBS) for 15 min at 37°C. The fluorescence images were recorded and quantified by using a fluorescence microscope (Leica Microsystems) with excitation at 540 nm and emission at 590 nm.

### 2.7. Measurement of Intracellular ROS Accumulation

To monitor the intracellular accumulation of ROS, the fluorescent probe DCF-DA was used. After treatment of SH-SY5Y cells (8 × 10^4^ cells/500 *μ*L in 4-well chamber slide) with A*β*_25–35_ (15 *μ*M) in the presence or absence of SUL for 6 h, cells were incubated with DCF-DA solution (50 *μ*M in PBS) at 37°C for 15 min. The fluorescence signals inside cells were excited at 488 nm and emission was monitored at 535 nm. The images were recorded with a fluorescence microscope (Leica Microsystems).

### 2.8. Protein Oxidation

The levels of protein carbonyls were determined by using OxyBlot Protein Oxidation Detection Kit (Millipore, MA, USA) according to the manufacturer's instruction. Briefly, the protein samples (15 *μ*g) were denatured by SDS (6% final concentration) and then derivatized to 2,4-dinitrophenylhydrazone (DNP-hydrazone) by incubation with 2,4-dinitrophenylhydrazine (DNPH) for 15 min at RT. After adding neutralization solution, the samples were electrophoresed on a 10% SDS-PAGE gel and transferred to PVDF membrane. The membrane was incubated with blocking buffer for 1 h to reduce nonspecific binding and then reacted with anti-DNP primary antibody for 1 h at RT. After two times washing with PBST, the membrane was further incubated with HRP-conjugated secondary anti-rabbit antibody for 1 h at RT. The carbonylation bands were detected by using chemiluminescence method (Pierce Biotechnology).

### 2.9. Nuclear Protein Extraction

Nuclear protein extracts were prepared by using the Nuclear Extraction Kit (Chemicon, Inc., MA, USA). After treatment, SH-SY5Y cells were washed with ice cold PBS and harvested by centrifugation. The harvested cells were resuspended in ice-cold cytoplasmic lysis buffer, incubated on ice for 15 min, and centrifuged at 8,000 g for 20 min at 4°C. The pellet was resuspended in ice-cold nuclear extraction buffer, incubated on ice for 60 min using shaker, and centrifuged at 16,000 g for 7 min at 4°C. The supernatant containing nuclear proteins were stored at −80°C for western blot analysis and electrophoretic mobility shift assay (EMSA). Protein concentrations were determined by Bradford assay (BIO-RAD, CA, USA).

### 2.10. Electrophoretic Mobility Shift Assay (EMSA)

The DNA binding activity of Nrf2 to antioxidant response element (ARE) was assessed by LightShift Chemiluminescent EMSA kit according to the procedure provided from Pierce Biotechnology. The isolated nuclear protein samples were combined with binding mixture (1 *μ*g poly (dI·dC), 50% glycerol, 1% NP-40, 1 M KCl, 100 mM MgCl_2_, and 200 mM EDTA (Pierce Biotechnology)) and incubated on ice for 20 min. Subsequently, biotin-labeled oligonucleotide specific to Nrf2 (5′-TGGGGAACCTGTGCTGAGTCACTGGAG-3′, Panomics, CA, USA) was added to the reaction mixture and additionally incubated for 10 min at RT. The DNA-protein complexes were separated on the 6% nondenaturing polyacrylamide gel at 80 V for 1 h and then transferred to nylon membrane (Pall Co., MI, USA) at 380 mA for 45 min. The membrane was subjected to immediate cross-linking by transilluminator at 312 nm for 10 min. After blocking the membrane with blocking buffer for 15 min at RT, the membrane was incubated with stabilized streptavidin-HRP for 15 min at RT. After three times washing with wash buffer, the DNA-protein complex bands were detected by chemiluminescence method (Pierce Biotechnology).

### 2.11. Synthetic Small Interfering RNA (siRNA) Transfection

 For the knockdown experiments of Nrf2, SH-SY5Y cells were transiently transfected with siRNA of Nrf2 (Nrf2-siRNA) using DOTAP transfection reagent (Roche Diagnostics GmbH) in accordance with the manufacturer's protocol. The sequences of the sense and antisense strands of the human Nrf2-siRNA were as follows: 5′-AAG AGU AUG AGC UGG AAA AAC TT-3′    (sense) and 5′-GUU UUU CCA GCU CAU ACU CUU TT′-3′ (antisense) which were selected by siRNA Target Finder software provided by Invitrogen. After transfection of SH-SY5Y cells with Nrf2-siRNA, cells were further exposed to A*β*_25–35_ (15 *μ*M) for 24 h in the presence or absence of SUL (5 *μ*M) pretreatment and then cell viability and molecular markers for apoptotic cell death were examined.

### 2.12. Statistical Analysis

SPSS software 13.0 (SPSS, Inc, Chicago, IL, USA) was used for the statistical analysis. All data represent at least three independent experiments and are expressed as mean ± SD. Statistical comparisons between groups were made by one-way analysis of variance (ANOVA) followed by Tukey's test as *post hoc* analysis to determine individual group differences. Statistical significance was accepted at value of  *P*  less than 0.05.

## 3. Results

### 3.1. Protective Effect of SUL against A*β*_25–35_-Induced Cytotoxicity and Apoptotic Cell Death

**Figure 1:**
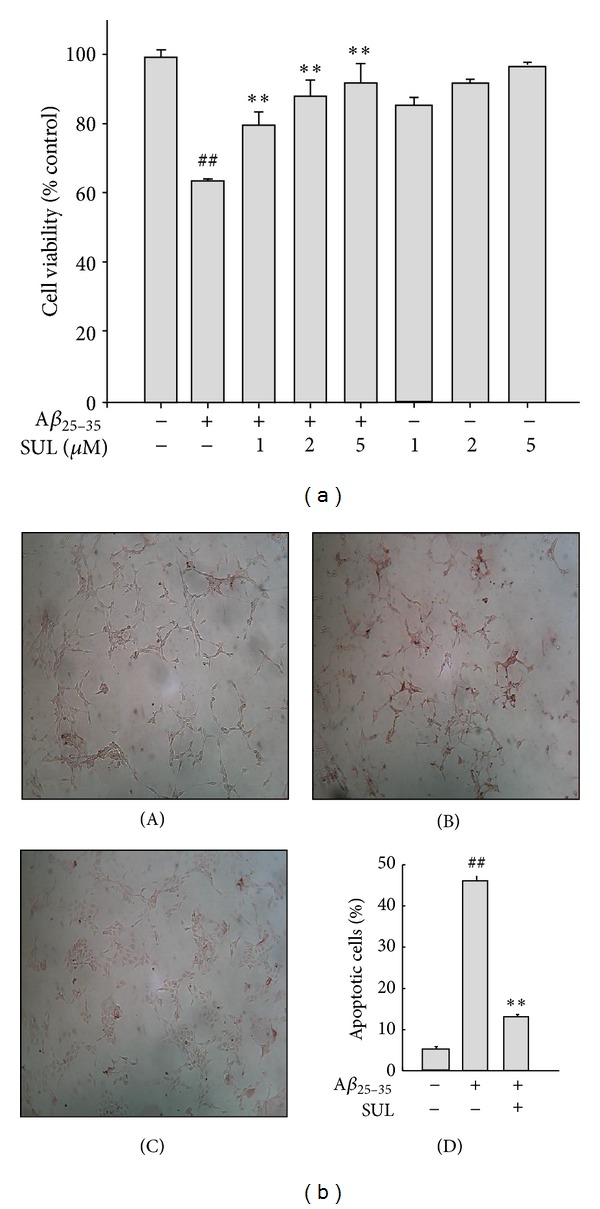
Protective effect of sulforaphane (SUL) on A*β*_25–35_-induced cytotoxicity and apoptotic cell death. SH-SY5Y cells were incubated with 15 *μ*M A*β*_25–35_ with or without SUL (1 *μ*M, 2 *μ*M, and 5 *μ*M) for 24 h. (a) Viable cells were determined by MTT dye reduction assay. Cell viability is expressed as the percentage of control. Data are represented as mean ± S.D. (*n* = 3). ^##^ *P* < 0.01, control versus A*β*_25–35_ and **P < 0.01, A*β*_25–35_  versus A*β*_25–35_ + SUL. (b) DNA fragmentation was measured by TUNEL. (A) Vehicle-treated control; (B) A*β*_25–35_ alone (15 *μ*M); (C) A*β*_25–35_ (15 *μ*M) + SUL (5 *μ*M); (D) quantification of apoptotic cell death (%).

We have investigated the effect of SUL on A*β*_25–35_-induced cytotoxicity and apoptotic cell death in SH-SY5Y cells by MTT dye reduction assay and TUNEL staining, respectively. Cells were incubated with various concentrations of SUL (1 *μ*M, 2 *μ*M, and 5 *μ*M) for 30 min followed by 15 *μ*M A*β*_25–35_ treatment for additional 24 h. Pretreatment of SUL protected against A*β*_25–35_-induced cytotoxicity in a concentration-dependent manner (Figure 1(a)). SUL-treated cells exhibited significantly higher cell viability than A*β*_25–35_-treated group did. In addition, A*β*_25–35_-induced apoptotic cell death was effectively suppressed by the pretreatment with SUL as assessed by TUNEL, which detects DNA fragmentation *in situ*, a typical marker for apoptosis (Figure 1(b)). SUL significantly reduced the number of TUNEL-positive cells caused by A*β*_25–35_  treatment.

**Figure 2:**
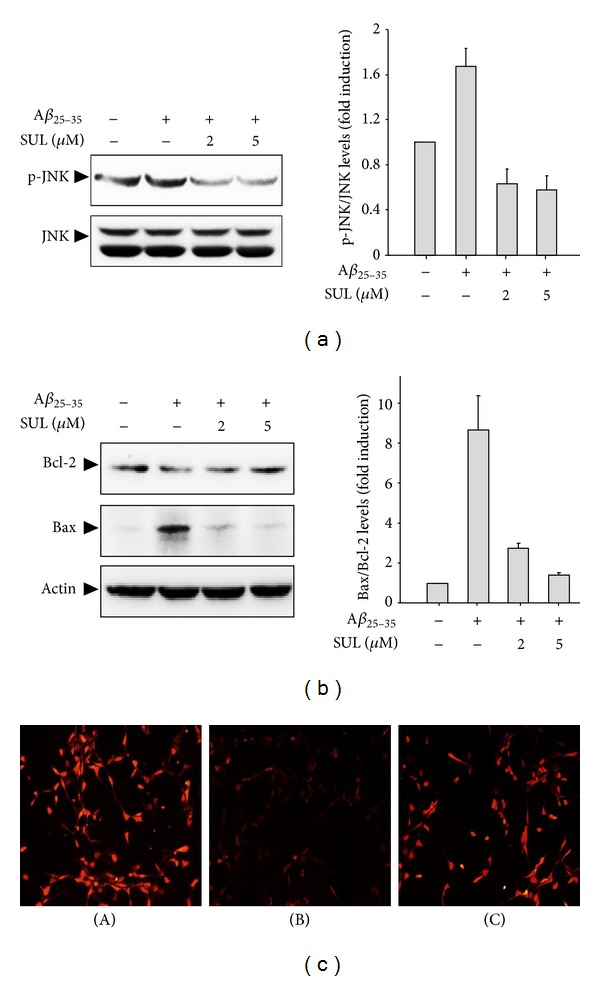
Protective effect of SUL on A*β*_25–35_-induced pro-apoptotic signals. SH-SY5Y cells were exposed to 15 *μ*M of A*β*_25–35_  in the presence or absence of SUL (2 *μ*M and 5 *μ*M) for 24 h. Activation of JNK (a) and expression of Bcl-2 family proteins (b) were assessed by western blot analysis using anti-phospho-JNK, anti-JNK, anti-Bcl-2, anti-Bax, and anti-actin antibodies. Actin levels were monitored to verify equal amount of protein loading. Relative expression levels of p-JNK/JNK and Bax/Bcl-2 were quantified from three independent experiments and are represented on the right panels. (c) Mitochondria membrane potential was measured by immunofluorescence staining using TMRE probe. The representative images of TMRE fluorescence were shown. (A) Vehicle-treated control; (B) A*β*_25–35_ alone (15 *μ*M); (C) A*β*_25–35_ (15 *μ*M) + SUL (5 *μ*M).

We also confirmed the protective effect of SUL against A*β*_25–35_-induced apoptotic cell death by examining pro- or antiapoptotic signals, such as activation of JNK, expression of Bcl-2 family proteins, and dissipation of mitochondrial membrane potential (MMP). A*β*_25–35_-induced apoptosis of SH-SY5Y cells was accompanied by activation of JNK via phosphorylation (Figure 2(a)) and a decreased Bcl-2 as well as an increased Bax protein levels (Figure 2(b)). However, pretreatment of SUL dramatically reduced A*β*_25–35_-elevated phosphorylation of JNK and expression of pro-apoptotic protein Bax. Moreover, anti-apoptotic protein Bcl-2 levels were effectively upregulated by SUL pretreatment. A*β*_25–35_  treatment also led to disruption of MMP as assessed by using TMRE cationic probe, which was shown as low fluorescence intensity compared with control group (Figure 2(c)). However, SUL pretreatment effectively restored A*β*_25–35_-decreased TMRE fluorescence intensity up to control levels representing recovery from the dissipation of MMP.

### 3.2. Inhibitory Effect of SUL on A*β*_25–35_-Induced ROS Production and Subsequent Oxidative Damages

**Figure 3:**
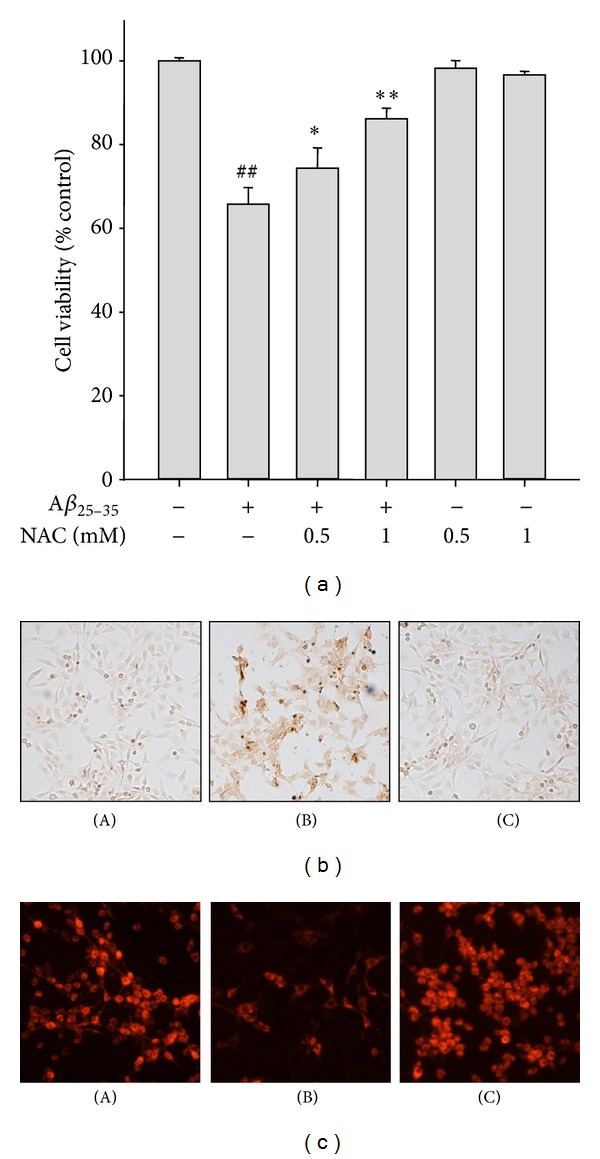
Protective effect of NAC on A*β*_25–35_-induced apoptotic cell death. SH-SY5Y cells were treated with A*β*_25–35_ (15 *μ*M) with or without NAC (0.5 mM and 1 mM) for 24 h. (a) Cell viability was measured and calculated by MTT dye reduction assay. Data are shown as mean ± S.D. (*n* = 3). ^##^ *P* < 0.01, control versus A*β*_25–35_ and  *P < 0.05 or  **P < 0.01, A*β*_25–35_ versus A*β*_25–35_ + NAC. (b) Apoptotic cell death was examined by TUNEL staining. (A) Vehicle-treated control; (B) A*β*_25–35_ alone (15 *μ*M); (C) A*β*_25–35_ (15 *μ*M) + NAC (1 mM). (c) MMP was monitored by relative TMRE fluorescence intensity. (A) Vehicle-treated control; (B) A*β*_25–35_ alone (15 *μ*M); (C) A*β*_25–35_ (15 *μ*M) + NAC (1 mM).

**Figure 4:**
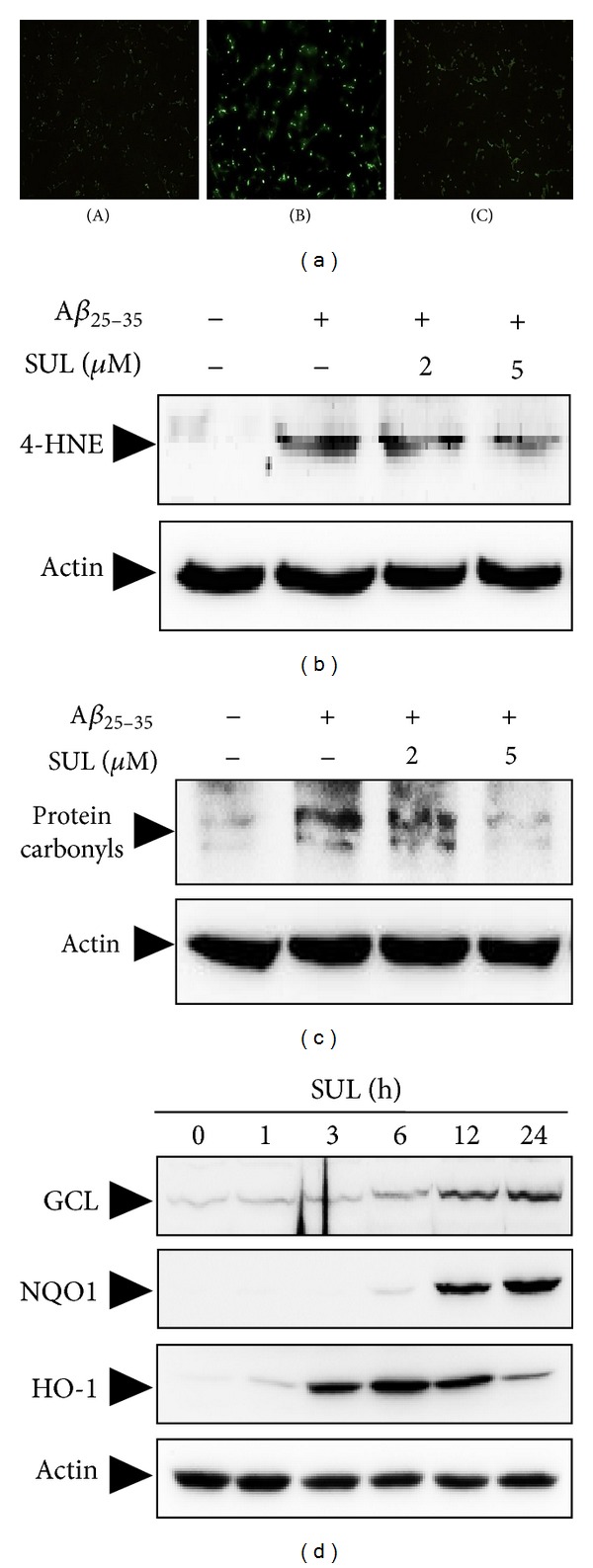
Inhibitory effect of SUL on the A*β*_25–35_-induced intracellular accumulation of ROS and oxidative stress. (a) SH-SY5Y cells were treated with 15 *μ*M A*β*_25–35_ for 6 h with or without SUL pretreatment for 30 min. Intracellular ROS levels were monitored by using DCF-DA fluorescence dye. (A) Vehicle-treated control; (B) A*β*_25–35_ alone (15 *μ*M); (C) A*β*_25–35_ (15 *μ*M) + SUL (5 *μ*M). ((b)-(c)) SH-SY5Y cells were exposed to 15 *μ*M A*β*_25–35_ in the presence or absence of SUL (2 *μ*M and 5 *μ*M) for 24 h. Molecular markers for oxidative damages such as lipid peroxidation (b) and protein oxidation (c) were determined by western blot analysis and protein carbonyl assay as described in Section 2. (d) The protein expression of antioxidant enzymes for example GCS, NQO-1, and HO-1 was evaluated by western blotting using their specific antibodies. Actin levels were assessed to confirm the equal amount of protein loaded.

It has been reported that A*β*_25–35_-induced cytotoxicity and apoptotic cell death are mediated by oxidative stress. In another experiment, A*β*_25–35_-induced cytotoxicity (Figure 3(a)) and apoptotic cell death such as DNA fragmentation (Figure 3(b)) and impairment of MMP (Figure 3(c)) were effectively suppressed by pretreatment with NAC (0.5 mM and 1 mM), a glutathione (GSH) precursor with strong antioxidant activity. Based on the involvement of oxidative stress in A*β*_25–35_-induced apoptosis in SH-SY5Y cells, in the next experiment we have examined the effect of SUL on A*β*_25–35_-induced ROS formation. Cells were pretreated with SUL for 30 min before incubation with A*β*_25–35_ (15 *μ*M) for additional 6 h. A*β*_25–35_ treatment led to intracellular accumulation of ROS, which was attenuated by SUL pretreatment (Figure 4(a)) as assessed by relative fluorescence intensity of DCF-DA dye. The results indicated that SUL could inhibit A*β*_25–35_-induced ROS production in SH-SY5Y cells.

It is well known that ROS can cause oxidative stress to critical cellular macromolecules such as DNA, protein, and lipids. In the present study, treatment of A*β*_25–35_ (15 *μ*M) caused oxidative damages to lipids (Figure 4(b)) and proteins in SH-SY5Y cells (Figure 4(c)), which were measured by formation of 4-HNE and protein carbonyls, respectively. 4-HNE and protein carbonyls are indicators of oxidative stress and key markers for oxidation of lipid and protein. A*β*_25–35_-induced lipid peroxidation (Figure 4(b)) and protein oxidation (Figure 4(c)) were substantially reduced by pretreatment of these cells with SUL.

### 3.3. Augmentation of Cellular Antioxidant Defense Capacity by SUL via Activation of Nrf2

To investigate molecular mechanisms of neuroprotection exerted by SUL against A*β*_25–35_-induced oxidative cell death, we have assessed expression levels of cellular antioxidant enzymes such as GCS, NQO-1, and HO-1. SH-SY5Y cells were treated with 5 *μ*M SUL for the indicated time periods, and protein levels of GCS, NQO-1, and HO-1 were determined by western blot analysis using specific antibodies. As shown in Figure 4(d), the expression of GCS and NQO-1 was increased by SUL treatment in a time-dependent manner which peaked at 24 h. In addition, HO-1 protein levels increased from 3 h after SUL treatment and were maintained up to 12 h (Figure 4(d)). All these results indicated that SUL could induce the expression of antioxidant enzymes to protect cells from oxidative damages caused by A*β*_25–35_ in SH-SY5Y cells.

**Figure 5:**
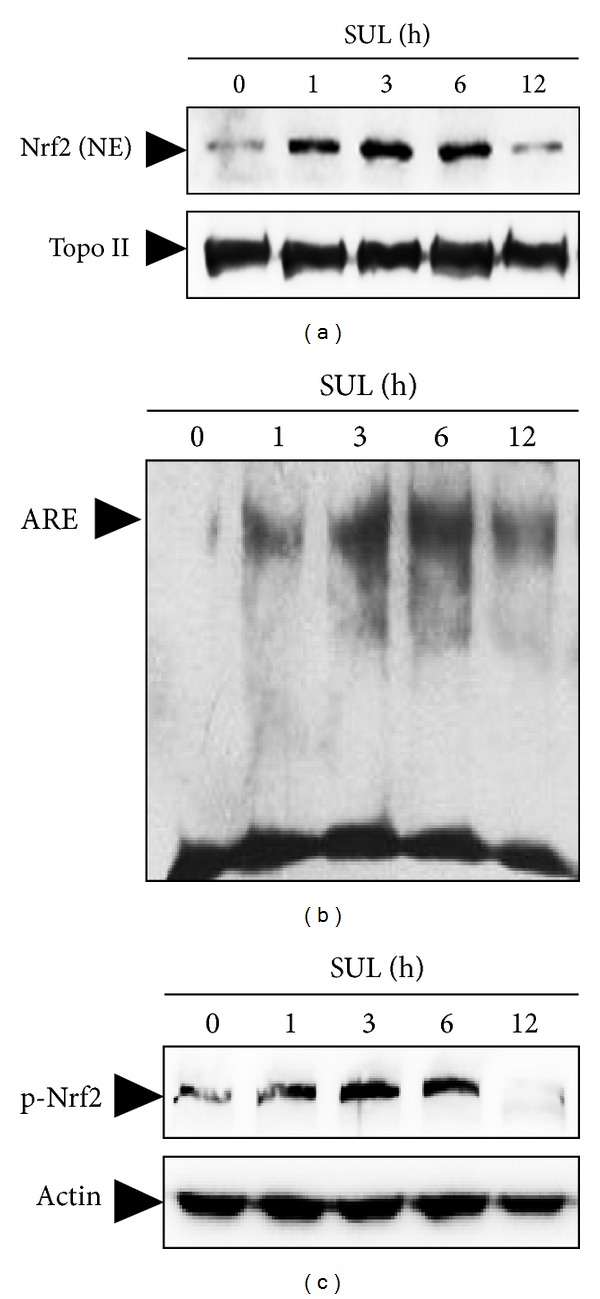
SUL-induced activation of Nrf2 in SH-SY5Y cells. Cells were incubated with SUL (5 *μ*M) for indicated times and nuclear as well as total protein sample extracts were prepared. (a) Nuclear translocation of Nrf2 was monitored by western blotting of nuclear extracts by probing with anti-Nrf2 specific antibody. (b) Nrf2-ARE binding activity was measured by EMSA according to the manufacturer's instruction using biotin-labeled oligonucleotide specific for Nrf2. (c) Phosphorylation of Nrf2 was assessed by western blot analysis with anti-phospho-Nrf2 (Ser40) antibody. The levels of Topo II (a) and actin (c) were examined to ensure equal amount of nuclear and total protein as loading controls, respectively.

To elucidate upstream regulator for the SUL-induced up-regulation of the antioxidant enzymes, we have focused on the activation of redox-sensitive transcription factor Nrf2. When SH-SY5Y cells were treated with 5 *μ*M SUL for the indicated times, nuclear translocation (Figure 5(a)), ARE-DNA binding (Figure 5(b)), and phosphorylation (Figure 5(c)) of Nrf2 were assessed by western blot analysis and EMSA. Treatment of SH-SY5Y cells with SUL increased nuclear levels of Nrf2 (Figure 5(a)) and Nrf2 binding to ARE promoter sequence (Figure 5(b)) with similar kinetic patterns. Moreover, SUL treatment increased phosphorylation of Nrf2 at Ser-40 residue as well (Figure 5(c)), which is known to facilitate the dissociation of Nrf2 from Keap1 rendering its translocation to nucleus.

**Figure 6:**
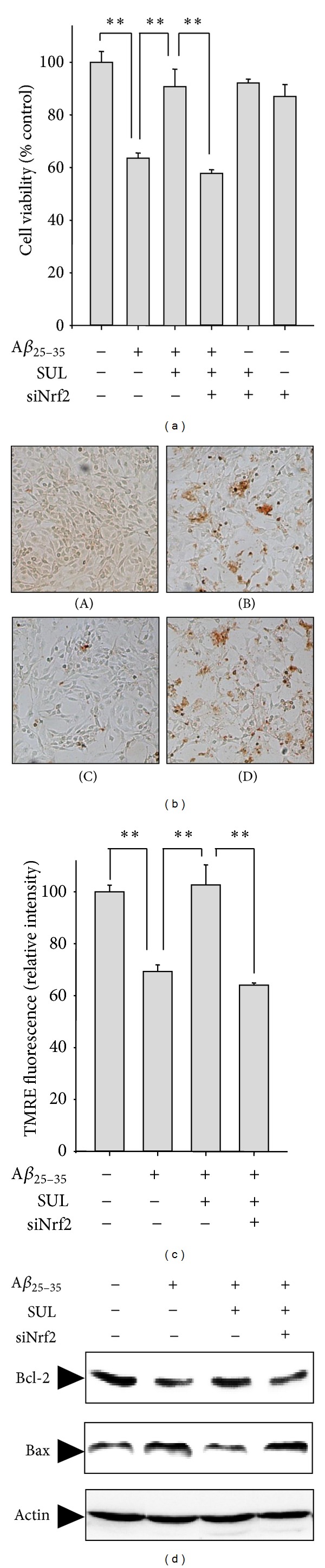
Effect of Nrf2 gene knock-down on SUL-mediated protection against A*β*_25–35_-induced apoptotic cell death. SH-SY5Y cells were transiently transfected with siRNA of Nrf2 according to the protocol provided by manufacturer and then exposed to A*β*_25–35_ (15 *μ*M) in the presence or absence of SUL (5 *μ*M) for 24 h. (a) MTT assay was performed to measure cell viability. Data are represented as mean ± S.D. (*n* = 3). **P < 0.01, significantly different between groups. (b) TUNEL staining was conducted to verify DNA fragmentation *in situ*. (A) Vehicle-treated control; (B) A*β*_25–35_ alone (15 *μ*M); (C) A*β*_25–35_ (15 *μ*M)  + SUL (5 *μ*M); (D) A*β*_25–35_ (15 *μ*M) + SUL (5 *μ*M) + Nrf2-siRNA. (c) TMRE staining was performed to compare MMP. Data are represented as mean ± S.D. (*n* = 3). **P < 0.01, significantly different between groups. (d) Protein expression of Bcl-2 and Bax was determined by western blot analysis using specific antibodies. Actin levels were examined to ensure equal amount of protein loading.

To further verify the direct role of Nrf2 in mediating the cytoprotective effect of SUL against A*β*_25–35_-induced oxidative cell death, we have downregulated the Nrf2 expression by transient transfection of SH-SY5Y cells with Nrf2-siRNA. The cellular protection of SUL on A*β*_25–35_-induced cytotoxicity (Figure 6(a)) and DNA fragmentation (Figure 6(b)) were abolished by knockdown of Nrf2 gene with Nrf2-siRNA. Moreover, the protective effect of SUL on A*β*_25–35_-mediated proapoptotic signals such as decreased MMP (Figure 6(c)) and increased Bax/Bcl-2 ratio (Figure 6(d)) and subsequent oxidative damages to lipids determined by 4-HNE formation (data not shown) were substantially abrogated by Nrf-siRNA transfection. These results suggest a critical role of Nrf2 in SUL-mediated protection against A*β*_25–35_-induced apoptotic cell death.

## 4. Discussion

In this study, we have examined the protective effect and molecular mechanism of SUL against A*β*-induced oxidative and apoptotic cell death. The results from the MTT assay and apoptotic analysis (TUNEL) provided a direct evidence demonstrating that SUL could protect SH-SY5Y cells from A*β*_25–35_-induced toxicity through increasing cell viability as well as inhibiting the apoptotic cell death. We also have assessed the effect of SUL on the A*β*_25–35_-induced pro-apoptotic signals such as activation of JNK and increased ratio of Bax to Bcl-2. Pretreatment of SUL elevated the anti-apoptotic Bcl-2 protein levels, decreased the pro-apoptotic Bax protein expression, and attenuated JNK activation via inhibition of its phosphorylation. 

It has been reported that A*β*_25–35_-induced cytotoxicity was mediated by oxidative stress. The excessive production of ROS by A*β*_25–35_ and exhaustion of the endogenous antioxidant defense system including GSH, catalase, superoxide dismutase, and glutathione metabolizing enzymes can cause oxidative damages to critical cellular macromolecules, mitochondrial dysfunction, and altered cellular signal transduction cascades. In the present study, A*β*_25–35_ treatment led to intracellular accumulation of ROS in SH-SY5Y cells, which was effectively inhibited by pretreatment with SUL. Moreover, SUL could alleviate A*β*_25–35_-induced oxidative damages including formation of 4-HNE and protein carbonyls through decreasing ROS production. Dissipation of MMP reflects the opening of the mitochondrial permeability transition pore due to the ROS release from mitochondria [19]. In this study, during the apoptotic cell death induced by A*β*_25–35_, MMP generated by the gradient of ion concentrations between two sides of the mitochondrial membrane was decreased, whereas SUL pretreatment restored the dissipation of MMP. In accordance with our finding, it has been reported that SUL increases the resistance of liver mitochondria to redox-regulated permeability transition pore opening and elevates expression of antioxidant proteins involved in mitochondrial defense against oxidative stress [20].

As the accumulation of ROS can trigger imbalance of redox state, neuronal cells have a set of antioxidant defense enzymes that maintain homeostasis between them. Therefore, one way to render neuronal cells more resistant to A*β*-induced oxidative cell death is to potentiate the endogenous antioxidant defense system, for instance, to up-regulate an array of antioxidant enzymes. In the present study, treatment of SUL elevated the protein expression of antioxidant enzymes such as GCL, NQO-1, and HO-1. GCL is a rate-limiting enzyme for biosynthesis of GSH which is a representative endogenous antioxidant molecule and plays an important role in cellular defense against oxidative stress [21]. Because homeostasis of GSH and GSH-dependent enzymes are considered to be key determinants of antioxidant protection, dysregulation of GSH-related antioxidant network might bring about the initiation and progression of neurodegenerative diseases where oxidative stress is one of critical causes [22]. 

NQO-1 is a cytosolic flavoprotein that catalyzes the two-electron reduction of quinones to the redox-stable hydroquinones, preventing their redox cycling and eventually generating the ROS [23]. Increasing evidence supports the role of NQO-1 as a safety valve to sequester ROS and prevent severe oxidative damages in various neuronal disorders including AD [23, 24]. HO-1, known as heat shock protein 32, plays a crucial role in endogenous defense against oxidative stimuli-induced brain injuries by decomposing toxic heme into carbon monoxide, iron, and biliverdin [25]. Biliverdin is subsequently converted into bilirubin through the action of biliverdin reductase and these two molecules serve as potent radical scavengers protecting cells from oxidative damages. The pharmacological up-regulation of HO-1 expression in brain regions showed promising therapeutic effects in the models of neurodegenerative diseases and brain infections [26].

To further elucidate the upstream regulators for the induction of endogenous antioxidant defense enzymes against oxidative stress, we have focused on the Nrf2-ARE signaling pathway. Recently, abundant evidence suggests the protective functions of Nrf2 and Nrf2-regulated gene products in diverse neuronal disorders [27, 28]. Considering that Nrf2 mediates general antioxidant responses, Nrf2 could be a potential therapeutic target for neurodegenerative diseases, where cells are suffering from chronic state of oxidative stress. Under normal quiescent state, Nrf2 is sequestered in the cytoplasm by a cytoskeletal associated specific negative regulator, Kelch-like ECH associating protein 1 (Keap1). Upon exposure to ROS or xenobiotics, Nrf2 is liberated from Keap1, translocates from the cytosol to the nucleus, heterodimerizes with accessory proteins such as small Maf protein family, and sequentially binds to antioxidant response element (ARE) promoter region. The binding of Nrf2 to ARE induces the production of diverse antioxidant enzyme and phase II detoxifying genes such as GCL, glutathione-*S*-transferase (GST), UDP-glycosyltransferases (UGTs), HO-1, and NQO1, which protect cells against oxidative stress as well as a wide range of other toxins [27, 28].

In the present study, the cytoprotective effect of SUL against A*β*_25–35_-induced oxidative damage and cell death seemed to be mediated by up-regulation of antioxidant enzymes through Nrf2 activation. SUL has been considered as an indirect antioxidant because of its ability solely to induce many cytoprotective antioxidant enzymes through the Nrf2-ARE pathway [29]. Induction of the Nrf2-ARE pathway by SUL has been reported to prevent cytotoxicity caused by oxygen and glucose deprivation [15, 30], 6-OHDA [16, 31], superoxide [17], H_2_O_2_ and glutamate [18], 5-S-cysteinyl-dopamine [32], or A*β*_1–42_ [33] in neuronal cell lines as well as primary cultures. Furthermore, activation of the Nrf2-ARE pathway is able to protect against brain injuries in the animal models of neurodegenerative diseases [34, 35], spinal cord injury [36–38], focal cerebral ischemia [39], hypoxia-ischemic injury [14], traumatic brain injury [40], subarachnoid [41] or intracerebral hemorrhage [42], or epilepsy [43]. According to *in vivo* studies, strategies to potentiate Nrf2-ARE pathway by SUL were proved to be useful in improving memory impairment and cognitive dysfunction caused by traumatic brain injury [44] or A*β* [34]. Conversely, Nrf2 KO mice models of neurological disorders including Parkinson's disease [35], spinal cord injury [37], traumatic brain injury [40], intracerebral hemorrhage [42], and epilepsy [43] exhibited increased susceptibility to neurological oxidative damages but did not maintain any benefits from the protective effects of SUL. In our experiment, the protective effect of SUL against A*β*_25–35_-caused apoptotic cell death was abolished by down-regulation of Nrf2 gene by transient transfection with Nrf2-siRNA.

Although the molecular milieu of SUL-induced Nrf2 activation in SH-SY5Y cells has not been elucidated, two possible mechanisms for the activation of Nrf2-ARE pathway by SUL have been proposed in other types of cells. One is structural change of Keap1 due to the modification of specific cysteine residues by binding of SUL [29, 45]. The other is the phosphorylation of Nrf2 at Ser-40 residue by mitogen-activated protein kinases [46], protein kinase C [46], and phosphatidylinositol 3-kinase/Akt activated by SUL [31, 47, 48]. Nrf2 phosphorylation by aforementioned kinases triggers the release of Nrf2 from inhibitory Keap1, thereby facilitating the Nrf2 translocation to nucleus. However, phosphorylation of Nrf2 at Tyr-568 residue by GSK-3*β* can promote its nuclear exclusion or proteolysis [46, 49]. Nevertheless, the molecular signaling pathways activating Nrf2 appears to be pleiotropic and dependent on cell types as well as stimuli.

## 5. Conclusions

**Figure 7:**
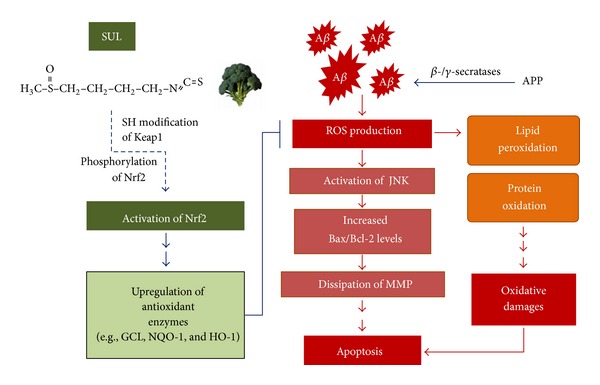
Schematic diagram that describes neuroprotective effects of SUL against A*β*-induced oxidative cell death in AD. SUL attenuates A*β*-induced oxidative damages, pro-apoptotic signals, and apoptotic cell death through the activation of Nrf2-ARE signaling pathway, which consequently fortify Nrf2-dependent antioxidant defense capacity.

In conclusion, a phytochemical SUL attenuates A*β*_25–35_-induced oxidative stress and pro-apoptotic signals such as activation of JNK, an increase in pro-apoptotic Bax, and a decrease in anti-apoptotic Bcl-2, thereby inhibiting apoptotic neuronal cell death in SH-SY5Y cells. Moreover, SUL induced the activation of Nrf2-ARE signaling pathway, which consequently results in up-regulation of Nrf2-dependent antioxidant capacity, leading to reduction in the A*β*_25–35_-induced oxidative damages (Figure 7). Taken together, the results in the present study suggest pharmacologic activation of the Nrf2 signaling pathway by SUL might be a practical preventative and therapeutic strategy for AD patients. However, further studies are required to obtain more insights into the molecular mechanisms of SUL-induced Nrf2 activation and clinical application of SUL.
